# Report of Clinical Observations on Continued (Typhus and Typhoid) Fever

**Published:** 1850-12

**Authors:** Austin Flint

**Affiliations:** Prof. of the Principles and Practice of Medicine, and Clinical Medicine, in the University of Buffalo


### BUFFALO MEDICAL JOURNAL

AND

### MONTHLY REVIEW.

VOL. 6. DECEMBER, 1850. NO. 7.

### ORIGINAL COMMUNICATIONS.

---

ART. I.—*Report of Clinical Observations on Continued (Typhus and Typhoid) Fever. Based on an Analysis of Fifty-Two Cases.* By Austin Flint, M. D., Prof. of the Principles and Practice of Medicine, and Clinical Medicine, in the University of Buffalo.

(*Continued from page* 346.)

SECTION EIGHTH.

*Symptoms referable to the Circulation. Pulse, Capillary Congestion.* The points of interest in this section, relate, for the most part, to the pulse, and the circulation in the capillary vessels of the cutaneous surface. As respects the pulse, the question arises, how shall the facts contained in the histories be presented? In frequency, and other characters of the pulse, no two of the cases are precisely alike, and, therefore, they cannot be enumerated in classes. Moreover, in individual cases, the pulse was in no instance entirely uniform throughout the career of the disease, and frequently varied, not only daily, but at different periods of the same day. In order to exhibit, as concisely and comprehensively as practicable, the facts which the histories contain, the readiest and best method is to arrange them in a tabular form. I shall adopt this method, assigning a distinct table, respectively, to the cases of *Typhoid, Typhus,* and those of *doubtful type.* These tables will, in general, embrace all the facts with respect to the circulation embraced in the histories. To this there are few exceptions, arising from an omission to include all the details in the preliminary analysis. In the private cases, it will be observed, that the facts are expressed in general terms, with less regard to details and precision. This arises from the symptoms in these cases not having been noted daily at the bed side, as has been before stated. In the hospital cases, usually, the frequency and character of the pulse, etc., were daily recorded, but to this rule there were occasional exceptions. Generally speaking, so soon as convalescence was declared, this particularity in recording was no longer continued. In the majority of cases the pulse, etc., as well as other classes of symptoms, were only noted once daily, generally in the forenoon. The histories, therefore, do not embrace all the variations which may have occurred during the day, except in some instances. When this is the case, in the tables which follow, the letters A. M. and P. M., are attached to the figures denoting the frequency of the pulse. When these letters are not attached, it is understood that observations were made only in the forenoon; and the numbers following each other, without these letters, express the frequency of the pulse on different days.

The foregoing tables suffice to show, in the first place, that there exists considerable diversity in the frequency, and other characters of the pulse both in different cases of Continued Fever and in diff rent periods of the febrile career in individual cases.

It is probably never the case that the pulse remains wholly unaffected throughout the febrile career. A near approximation to this is apparently presented in the table of Typhoid hospital cases, No. 13. In this case the pulse was but 72, except on one day, when it was 96—the mean frequency being 76. This patient, however, had been confined to the bed eleven days before entering the hospital, during which time it is altogether probable that the pulse had been more or less accelerated.

The numbers given in the tables do not furnish a perfectly accurate representation of the condition of the pulse, since, generally, observations were made but once daily, and various transient causes are liable to affect the pulse at the time of the daily examination. To render this portion of the histories complete, the pulse should have been enumerated in each case several times daily, and the mean result taken, together with the variations.

The variations which in most of the cases are apparent on different days, in part, doubtless, denote fluctuations incident to the progress of the disease; but they are also, in part, to be referred to extrinsic circumstances affecting the condition of the patient, among which are to be included the influences of remedies.

On comparing the frequency of the pulse in the two types, *Typhus* and *Typhoid*, it is found that, it is considerably greater in the majority of cases of the former than of the latter. The average mean in *thirteen* of the hospital cases of *Typhoid* is 95 7-13 ; while in *nine* cases of *Typhus*, the average is 105 4-9. The fatal cases are excluded from this calculation. Greater frequency of the pulse, then, characterizes Typhus, a result which accords with the observations of others. Instances of a high maximum are oftener presented in *Typhus*. In the table of *Typhus* cases, exclusive of the cases that proved fatal, the pulse had a maximum of 140 in two cases in which recovery took place, and it was 136 in another case. In the cases of Typhoid, on the other hand, the highest maximum was 128, and this was attained in but a single case. Two of the fatal cases presented a greater frequency, 160.

An examination of the tables will also show that instances of feebleness, and irregularity of the pulse occurred oftener in cases of *Typhus* than in *Typhoid*.

In general terms, I should say, that the frequency of the pulse is a pretty good criterion of the severity of the disease. This opinion, however, is not based on a careful examination of all the cases in order to compare the pulse, as regards frequency, with the grade of severity. This would require not a small degree of labor. But if this be a general rule, it is not without exceptions, for, in some of the cases in my collection, the pulse numbered 120, when, in other particulars the disease was mild, and milder than in other cases in which the frequency of the pulse was less. It did not exceed 120, the day before the decease in one of the fatal cases, and in another, in so far as data were preserved, it fell below this number. In a case which has recently fallen under my observation, not included in this collection, the pulse, two days before the fatal issue, was but slightly accelerated. There can be no doubt if the pulse exceed 120, except for a transient period, that the case is of dangerous severity ; and the danger increases in more than a geometrical ratio if it rise above this point.

A deception occurred in one of the cases of *Typhus* (No. 6) which demands a passing notice. The pulse had been daily noted as "small," or "very small," and, on one day, "scarcely appreciable." This symptom, although in other respects the disease did not appear to be of a severe grade of intensity, was regarded as denoting danger, and stimulants were directed more freely than would have been the case had not the pulse presented this character. On the ninth day, I discovered that there was a bifurcation of the radial artery, one branch pursuing the usual course, and the other winding over the radius, the former being much the smaller of the two. From the position of the bed, I had always examined the pulse in the arm in which this peculiarity existed. The same anomaly I have met with in several other instances. The neglect to discover it in this instance affecting materially the treatment, may serve to suggest the importance of looking for a similar explanation in cases in which smallness and feebleness of the pulse are marked.

It will be observed on reference to the tables, that a few cases presented a minimum in the number of the pulse considerably below the normal average. In No. 16 of the hospital *Typhoid* cases, the pulse, on one day, was noted 64. In No. 1 of the *Typhus* cases, it was, on one day, 52. In Mo. 1 of the cases of *doubtful type*, it was on one day 52. In all these cases this reduction in the number of the pulse occurred at the commencement of convalescence, and was of transient duration. In none of these cases was there reason to suppose that the pulse, in health, was habitually slower than the average. Marked slowness of the pulse, then, occasionally characterizes convalescence in Continued Fever. Oftener the pulse continued somewhat accelerated after convalescence, by other symptoms, was fully declared. Generally speaking, a return to a normal state, as respects frequency and other characters of the pulse, is one of the best criterions of a favorable termination of the disease.

With respect to *exacerbations of fever*, in a large proportion of the cases, observations having been made but once daily, the histories are silent. In some instances, however, it is noted that exacerbations were present, and in a few instances the data suffice to show that they were absent. The only conclusion to be drawn from these limited facts is, that they may, or may not occur during the career of Continued Fever. To determine the probability of their occurrence, statistics much more extensive than the present collection of cases furnishes, are requisite.

The presence of capillary congestion of the face, extremities, or entire surface of the body is noted, in the tables, among the symptoms referable to the circulatory system. This symptom, it will be perceived, is present in a much larger proportion of the cases of *Typhus* than in those of the *Typhoid* type. The pathological significancy of this symptom has already been commented on (section third.) On examination of the pulse in the cases in which this symptom is noted, in the tables, the fact already stated (page 32d) may be verified, viz., that it does not depend on causes affecting the general circulation, i. e., the circulation through the heart and arteries, since it does not sustain any relation to symptoms denoting that the forces carrying on the general circulation are compromised in a special manner. In other words, in the cases in which capillary congestion is noted, the pulse does not invariably denote an enfeebled or disordered circulation, but, on the contrary, in some of the cases in which the capillary congestion was marked, the arterial circulation was less disturbed, as indicated by the pulse, than in other cases in which that symptom was not present. The occasion of the capillary congestion is, therefore, obviously inherent in the capillary system, and, perhaps, in the blood itself. In the remarks on this point in section third, it was stated that pulmonary congestions are said to be more liable to occur in *Typhus* than in the *Typhoid* type of Continued Fever. This has been confirmed by the results developed in the preceding section. A comparison of these results with the greater frequency of capillary congestion of the surface in cases of *Typhus*, favors the supposition that the same condition of the capillary vessels, or of the blood, may affect internal organs ; and that the greater frequency of pneumonitis, in *Typhus*, is in this way susceptible of explanation.

SECTION NINTH.

*Symptoms (Exclusive of Eruptions) referable to the Skin.*

The Symptoms (exclusive of Eruptions) referable to the skin, may be distributed into those relating to *sweating* and *moisture*, to *dryness*, to *temperature*, to *congestive redness*, to *Erysipelas*, and to *gangrene*. The phenomena belonging to these several classes are commingled, more or less, in individual cases; and, hence, they are to be considered, not only under separate heads, but as they are presented together in combination, succession, or alternation, during the progress of fever. I will proceed, first, to inquiries respecting each of the above divisions distinctly, taking them up respectively in the order enumerated.

*Sweating and moisture.* In a considerable number of the cases of both types, sweating, more or less free or profuse, occurred either once, or oftener, during the febrile career. It was so noted in fourteen of *twenty-seven* cases of Typhoid, in which the histories contain information on this point. Of these *fourteen* cases, *seven* were in *private* practice, and *seven* were in *hospital*. These cases are exclusive of the instances in which sweating occurred either coincident with, or shortly before convalescence, or as a precursor of death. In a certain number of cases the termination of the disease, either fatally, or in convalescence, was characterized by this event, but as occurring under these circumstances it will be more appropriately considered in a subsequent section. In *eight* of the *fourteen* cases, sweating occurred more than once during the febrile career; in *seven* of the *eight* cases it occured several times, and in the remaining case twice. The periods of the disease at which it occurred, were varied; in fact, among these fourteen cases, each day of the febrile career would probably be represented. Perspiration in a lesser degree, constituting what was distinguished in the histories as *moisture* of the skin, occurred, in *ten* of the *twenty-seven* cases of Typhoid, viz.: in *four* of the cases in *private* practice, and in *six* of those in *hospital*. In these cases the skin was either moist almost constantly during the febrile career, or, generally, the moisture was present more or less frequently for hours, or days, in the course of the disease. It will be perceived, by the above enumerations, that sweating occurred in a larger proportion of the cases of *Typhoid*, than merely moisture of the skin—a curious fact, worthy of passing notice with a view to its possibly being found to possess some significancy in connection with other facts.

Sweating occurred in a proportion of the *Typhoid* cases in *private* practice larger then in those in *hospital*, being present in seven-ninths of the former, and in only four-ninths of the latter group. As this disparity is not susceptible of explanation by reference to difference in circumstances in the two groups, it probably shows that, in the proportion of instances in which this symptom is present in Typhoid Fever different collections of cases should not be expected to exhibit uniformity.

Of the *twelve* hospital cases of *Typhus*, sweating was noted in the histories of *five*. It occurred several times in *one* of these cases; once only, in *one* case; and *twice* in *two* cases. *Moisture* of the skin was noted in but *two* cases. Thus, as regards sweating, results do not show a marked difference, in the two types, in the frequency of its occurrence; but simply *moisture* of the skin was less frequently observed in *Typhus* than in *Typhoid*, occurring in the ratio of 1 to 6 in the former, and 1 to 3 in the latter.

Of the *eight* cases of *doubtful type* sweating was noted in *four*. Moisture is noted in a single case; and in one case the history is deficient in information respecting the symptoms referable to the skin.

Adding together the cases of the three groups above mentioned in which the condition of the skin was noted, the sum total is 46, and of this number sweating occurred in 23, i. e., in precisely one-half.

The sweating was not in all cases equal in degree; pains, however, were not taken to determine differences in this particular with exactness, which, it is obvious, would be difficult, if not impracticable. The terms *free*, *profuse*, *copious*, and *bathed in sweat*, were frequently employed in the histories to express the quantity of secreted fluid present on the surface, but in other histories it is simply stated that sweating had occurred, or was obobserved at the time of the examination. In *one* case of typhus it is stated that the hands were corrugated from the profuseness of perspiration. This has been styled the *washer-woman's sweat*, and has been thought to be a fatal sign. In the case in which it was noted the disease ended favorably. In *one* case of Typhus, in which sweating occurred twice; on the first occasion it was quite profuse, and limited to the forehead and face ; on the second occasion, it was copious on the face, and extended, but in a less degree, to the chest and upper extremeties. As a general remark the sweating during the career of the Fever was not attended, or immediately followed by any marked abatement of the severity of the disease, but, on the contrary, judged by the symptoms, the effect was the reverse. This may be enumerated as the rule, and I am not aware that there were any exceptions to it. No exceptions were noted in the preliminary analysis of the cases, and in several instances it is stated that this event was not connected with any improvement in the symptoms.

The foregoing results, it is believed, conflict with the ideas generally entertained by practitioners, respecting the frequency with which sweating occurs in Continued Fever, and the degree and the kind of importance to be attached to it, illustrate the influence of theoretical or preconceived notions in frustrating the lessons of experience, unless that experience be based on observations recorded and analyzed. It is a current belief that sweating generally proves salutary when it occurs in Continued Fever—a belief originating, probably, in the ancient humoral hypothesis of a specific morbid material being eliminated through the cutaneous secretions ; and apparently sustained by the instances in which this symptom is regarded as critical from its occurring concurrently with convalescence. Its correctness, however, is disproved by the facts which numerical investigations disclose, showing the large number of cases in which the event occurs, and, at different periods of the disease, without affecting, apparently, in a favorable manner, its character or progress; showing, moreover, as will be seen hereafter, that it occurs during the febrile career, without improvement, in a larger proportion of cases, than that in which it signalizes convalescence. If this view of the subject be correct, the propriety of pursuing measures, during the career of Continued Fever, to produce sweating is more than questionable.

It would be interesting to ascertain upon what ulterior conditions, or circircumstances, appertaining to Continued Fever, sweating is dependent. With reference to this point of inquiry, it would be important to establish any relations subsisting between this event, and other phenomena belongto the natural history of the disease. I have examined the histories of several of the cases characterized by sweating, in order to discover a clue to some connection of this kind, but without success. It occurs, not only at different periods of the disease, but apparently irrespective of the circulation, the temperature of the skin, and other symptoms. The antecedent morbid condition, or conditions, upon which it depends, and the circumstances involved in its production, are unknown. All that can be said of its causation is, that it is an event incident to the progress of Continued Fever, belonging in the category with other incidental events, such as acceleration of the pulse, coating of the tongue, somnolency, etc.—all of which are not more rationally explicable than it.

This fact should be mentioned,viz: in the majority of the instances in which sweating occurred, it took place during the night, or, commenced during the night, and was continued into the day. It is not noted in any of the histories that it was preceded by exacerbation of fever, but this may have been true of some cases without having been observed, the examination of the patients, and records of symptoms, being made, for the most part, in the morning, and the occurrence of exacerbations, therefore, not always ascertained.

It remains to inquire respecting the occurrence of sweating and moisture in the cases which ended fatally. In *nine* of the fatal cases included in the above enumerations, sweating was noted in *four*, a proportion, it will be observed, not far from that in which it is found in the cases terminating in recovery. Hence, this event has no particular bearing on the prognosis. Of these *nine* cases, *moisture* was noted in *three*, which is also not very far from the proportion which it bears in the cases which recovered. It is to be borne in mind, that in these enumerations, as well as in those which have preceded, sweating and moisture at, or toward the close of the career of the disease, are not included.

*Dryness.* The skin, if perfectly normal, is neither moist nor dry, but presents a medium state, conveying to the touch a sensation which is best described by applying to it, figuratively, the term *mellow*. It has been seen, that in Continued Fever, deviations from this condition are apt to occur in consequence of increased secretion from the sudorifarous glands, causing moisture, or sweating.

Deviations of an opposite kind may occur, in consequence of diminished secretion from these glands. The surface then communicates to the touch a sensation of dryness. Dryness of the skin, more or less, occurs in the great majority of the cases of Continued Fever. Of the *thirty* cases of *Typhoid*, I find but *four* in which it appears from the histories that this condition of the skin was entirely absent during the febrile career. This was true of but *one* of the *twelve* hospital cases of *Typhus*. In several of the cases, however, the records do not afford positive information on this point—viz: in *ten* cases of *Typhoid*, and *one* case of *Typhus*. This defect in the histories of these cases probably was owing to not always appreciating the importance of noting the presence of a symptom so familiar and almost universal as this. Had the skin remained moist, or mellow, throughout the disease, in any of the cases, I am quite sure the fact would have been embraced in the histories.

The occurrence of dryness was very variable, as respects the period or periods of the disease when it was observed; its duration; and also its relation to other conditions of the external surface. In five cases, it appeared early, and persisted through the greater part of the febrile career. In other cases, it was not observed until the disease had made more or less progress ; and, generally, it was present at intervals, alternating with other cutaneous symptoms. It was so irregular in these particulars, as also in its degree of intensity, that it would be difficult to condense, by means of classification, the facts contained in the histories of all the cases. The brief and very general account contained in the foregoing paragraph, will suffice for the historical portion of this division of the symptoms referable to the skin.

Dryness of the surface would seem to be a more *consistent* event in continued fever than moisture or sweating, since observations show that all the glandular secretions of the organism are diminished in this disease. It is, however, as little susceptible of rational explanation as the opposite conditions already considered. Like most of the phenomenal manifestations of fever, it is so connected with the primary, essential, morbid changes, in which the disease consists, that while these remain undiscovered, we can hardly hope to penetrate completely its causation. We can only study it in its various relations.

As a symptom, it obtains so invariably in continued fever of both types, and also in other affections, that it is of small or no value in diagnosis, and has very little pathological significance.

*Temperature.* Heat of the skin existed, more or less, in most of the cases. In a few instances, however, the temperature did not appear to be increased beyond what belongs to a state of health. The heat was associated with moisture, and, more rarely, with sweating ; but generally, dryness co-existed. In some cases the heat continued pretty steadily throughout the disease, and in other cases it occurred at intervals, and was of variable duration. That peculiar pungent or acrid heat called *Calor Mordicans*, which is regarded by some writers as characteristic of the *Typhus* type, was noted in but *two* cases, one of the *Typhus*, and the other of *doubtful* type.

The increase of temperature was of different gradations, from a slight elevation above the natural warmth, to a degree to which, in a hyperbolical sense, the term *burning* might be applied. Coldness of the surface was not noted in a single case.

Of temperature, as of dryness, it may be said, and for similar reasons, that the conditions of the skin in this respect are of little account in diagnosis, and have no special pathological significance.

When associated with dryness, heat may in part be explained by the diminished evaporation. It is not, however, wholly accounted for in this way, but it involves a positive increase in the disengagement of caloric, as is evidenced by the fact that it co-exists in some instances with moisture, and even sweating. To inquire concerning the rationale of its production under these circumstances, would be to travel beyond the limits of the facts before us.

As a general remark, applicable to the divisions previously considered, as well as to the subject of temperature, it may be said that in estimating the severity of the disease, and the prognosis, by the symptoms referable to the skin, no very definite or positive inferences are to be drawn from any of the deviations from the state of health, considered by themselves ; but a return or approximation to normal conditions, other things being equal, must, of course, be regarded as favorable.

*Gangrene.* Gangrenous ulceration of the skin occurred in but a single case, which was of the *Typhus* type. It appeared on the tenth day after the admission of the patient into hospital. The parts affected were the Sacrum, and a small space over the Scapula. In the latter situation the affection was slight, and, of short continuance. Over the Sacrum a considerable eschar followed, leaving a large and troublesome ulceration, which did not heal for several weeks after convalescence was pronounced. The patient, finally, completely recovered. This case in other respects did not exhibit unusual severity.

In a very few instances (three or four, as near as can be determined by recollection) some erythema over the Sacrum, or nates, was observed which disappeared on the application of astringent washes, and the use of air cushions, without proceeding further.

It has been noticed that in this disease, blistered surfaces are apt to degenerate into troublesome ulcerations. The opportunities afforded in the present collection of cases for verifying this point were extremely limited, vesication very seldom entering into the treatment. In the few instances in which blisters were applied, no such result followed.

Erysipelas did not occur in any case during the febrile career. It became developed in one case, succeeding Parotitis, after convalescence was established. It was situated on the face, and was treated by the application of ice to the part affected. It was of short duration, and did not attain a great degree of severity.

Having thus considered, under distinct heads, the several divisions of the symptoms referable to the skin, it remains to give some account of these symptoms as they were associated in individual cases. In many of the cases all the varieties of the foregoing cutaneous phenomena were presented, either in combination, succession, or alternation, during the career of the disease. Not only were variations observed in the same case on succeeding days, but uniformity frequently did not exist at different periods of the same day. Since the laws regulating these fluctuations are but little understood, and very little pathological or practical importanc belongs to them in the present state of knowledge, they are chiefly interesting as comprising a part of the natural history of the disease. The readiest method of exhibiting them as contained in the history of all the cases, would be to arrange them in a tabular form, in the mode adopted in other sections. This I have done for convenience of examination and reference in writing the foregoing remarks. It will, however, answer every purpose, and economize space, as well as the patience of the reader, to select a few cases from those of each type as illustrations, giving the symptoms referable to the skin, which were noted on each day during the career of the Fever. This plan will therefore be adopted.

*Typhoid.*

Case 1.—1st day, skin cool ; 2d, warm and moist ; 3d, cool, and bathed in perspiration ; 4th, warm and moist ; 5th, moist ; 6th, perspiring freely; 7th, perspiring profusely ; 8th, warm and mellow ; 9th, warm ; 10th, warm aud dry; 11th, perspiring copiously; 12th, warm and moist ; 13th, warm and mellow ; 14th, perspiring profusely ; 15th, warm and moist ; 16th, warm and mellow ; 17th, cool and moist ; 18th, cool and moist.

Case 2.—1st day, skin hot and dry ; 2d, dry and warm ; 3d, warm ; 4th, warm and mellow ; 5th, warm and dry ; 6th, warm and mellow. Deep congestive redness of face and extremities.

Case 3.—1st day, skin rather hot, perspired at night ; 2d, warm and mellow; 3d, hot and dry; 4th, warm and moist, and at night profuse perspiration ; 5th, rather hot and dry ; 6th, perspired at a. m., and profusely at p. m.; 7th, hot and bathed in perspiration, dry before decease, this case proving fatal.

Case 4.—1st day, skin hot and dry ; 2d, warm and moist ; 3d, hot and dry ; 4th, cool ; 5th, warm and mellow; 6th, warm ; 7th, warm and dry; 8th, moist ; 9th, warm and dry; 10th, warm ; 11th, warm and dry ; 12th, warm and dry ; 13th, warm ; 14th, warm and dry ; 15th, moist, 16th, warm and dry ; 17th, warm and dry ; 18th, warm and mellow. Congestive redness of the face.

### *Typhus.*

Case 1.—1st day, skin hot, and heat acrid ; 2d, cool ; 3d, normal temperature and dry; 4th, cool ; 5th, cool ; 6th, warm and very dry ; 7th, mellow ; 8th, cool ; 9th, copious perspiration, confined to forehead and face ; 10th, warm and dry ; 11th copious perspiration on face, extending in a moderate degree to the upper extremities, suderina, deep congestive redness, somewhat livid.

Case 2.—1st day, warm and moist ; 2d, dry, and temperature increased ; 3d, cool ; 4th, warm and mellow; 5th, mellow, temperature somewhat increased ; 6th, cool and moist ; 7th, cool and moist. Dingy congestion of face and extremities, at first considerable, gradually diminishing, but not disappearing entirely until after convalescence was pronounced.

Case 3.—2d day, cool and dry; 3d, noted that skin presented no change. Congestive redness. Fatal on the third day.

Case 4.—1st day, cool ; 2d, cool ; 3d, warm and dry; 4th, warm, and at p. m., hot ; 5th, moist ; 6th, moist and cool ; 7th, warm, and at p. m., moist.

### *Doubtful Type.*

Case 1.—1st day, hot and dry; 2d, hot and dry; 3d, warm and dry; 4th, warm ; 5th, dry and rather hot ; 6th, dry and rather hot ; 7th, face covered with perspiration, and remainder of the body dry; 8th, hot and dry ; 9th, hot and dry ; 10th, hot ; 11th, dry, but less burning heat ; 12th dry, not hot. Some congestive redness of face. This case proved fatal.

Case 2.—1st day, hot and dry ; 2d, dry, acrid heat, (calor mordicans;) 3d, warm ; 4th, moist, perspired freely during the night ; 5th, moist. Congestive redness of face. Parotiditis and erysipelas occurred, after convalesence was pronounced in this case.

The enumeration of days in the foregoing cases is dated from the time the patients came under observation.

(*To be Continued.*)

